# An explicit model to extract viscoelastic properties of cells from AFM force-indentation curves

**DOI:** 10.1016/j.isci.2022.104016

**Published:** 2022-03-05

**Authors:** Shada Abuhattum, Dominic Mokbel, Paul Müller, Despina Soteriou, Jochen Guck, Sebastian Aland

**Affiliations:** 1 Max Planck Institute for the Science of Light and Max-Planck-Zentrum für Physik und Medizin, Staudstr. 2, 91058 Erlangen, Germany; 2 Technische Universität Dresden, Biotechnology Center, Center for Molecular and Cellular Bioengineering, Tatzberg 47-51, 01307 Dresden, Germany; 3 Fakultät Mathematik und Informatik, Technische Universität Freiberg, 09599 Freiberg, Germany; 4 Fakultät Informatik/Mathematik, Hochschule für Technik und Wirtschaft Dresden, 01069 Dresden, Germany

**Keywords:** Biophysics, Biomechanics, Materials science

## Abstract

Atomic force microscopy (AFM) is widely used for quantifying the mechanical properties of soft materials such as cells. AFM force-indentation curves are conventionally fitted with a Hertzian model to extract elastic properties. These properties solely are, however, insufficient to describe the mechanical properties of cells. Here, we expand the analysis capabilities to describe the viscoelastic behavior while using the same force-indentation curves. Our model gives an explicit relation of force and indentation and extracts physically meaningful mechanical parameters. We first validated the model on simulated force-indentation curves. Then, we applied the fitting model to the force-indentation curves of two hydrogels with different crosslinking mechanisms. Finally, we characterized HeLa cells in two cell cycle phases, interphase and mitosis, and showed that mitotic cells have a higher apparent elasticity and a lower apparent viscosity. Our study provides a simple method, which can be directly integrated into the standard AFM framework for extracting the viscoelastic properties of materials.

## Highlights

• Simple mechanical model to describe viscoelastic properties of soft matter
• A model fitted directly to force-indentation curves
• Capturing the distinct nature of hydrogels crosslinked in different mechanisms
• Comparing viscoelastic properties of cells in interphase and mitotic states

## Graphical abstract

**Figure:**
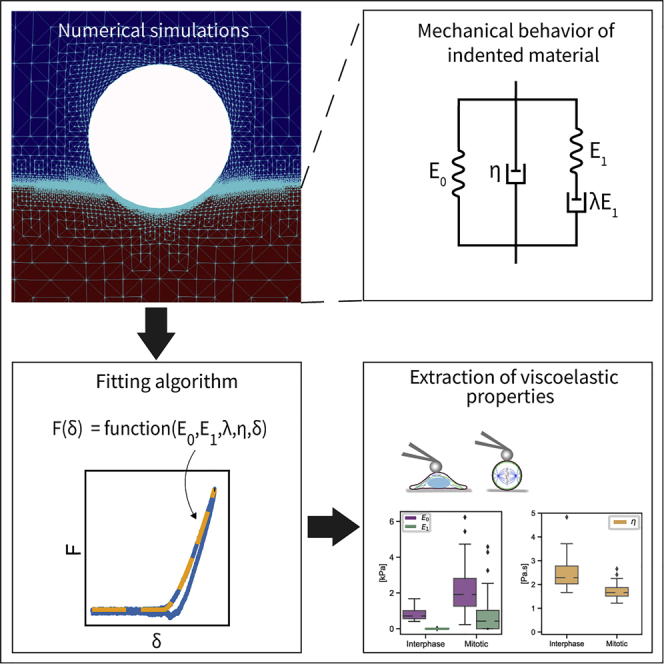

## Introduction

The viscoelastic behavior of soft materials, especially cells and tissues, has been extensively investigated due to its importance in many biological and physiological processes that take place during development and even disease (Lautenschläger et al., 2009; Diz-Muñoz et al., 2013; Darling et al., 2008). Many techniques are used to quantify the mechanical properties of cells, among them micropipette aspiration (Hochmuth, 2000), optical stretching (Guck et al., 2000), deformability cytometry (Otto et al., 2015), and atomic force microscopy (AFM) (Kuznetsova et al., 2007). The AFM, in particular, is still nowadays one of the most popular methods due to its conformity with various material types and geometries and the rather simple analysis process of the material properties. For a typical AFM indentation measurement, a cantilever, with a distinct tip shape, moves toward the sample with a predefined velocity and indents it until a prescribed force is reached. The cantilever then moves upwards while detaching from the sample. The deflection and displacement signals of the cantilever are processed further to extract the mechanical properties of the sample. Generally, a Hertzian model is fitted to the approach part of the force-indentation curves to quantify the apparent Young's modulus (Hertz, 1881). When applying the Hertzian model, few assumptions need to be considered, such as the material being homogeneous, isotropic, and linearly elastic. Cells and tissues, however, show not only elastic but also viscous behavior that is evident from the hysteresis between the approach and retraction segments of the force-indentation curve. Consequently, assessing this viscoelastic behavior is imperative for understanding the complex nature of biological matter (Fabry et al., 2001; Rother et al., 2014).

A number of studies utilized AFM to measure the viscoelastic properties of cells in both time and frequency domains (Darling et al., 2007, 2008; Rother et al., 2014; Alcaraz et al., 2003; Moreno-Flores et al., 2010; Mokbel et al., 2020; Mahaffy et al., 2000; Garcia and Garcia, 2018; Benaglia et al., 2019). Ideally, to investigate the whole range of the viscoelastic behavior one needs to probe the material for a long time and observe its response or apply oscillatory signals and evaluate its phase lag. These approaches require the user to alter the probing method and add several steps to account for the time-dependent drift or the effect of the hydrodynamic drag of the surrounding medium. On top of that, in many of studies, the biological materials were probed with a linear approach followed by immediate retraction movement (Abuhattum et al., 2018; Escolano et al., 2021; Möllmert et al., 2020). The force-indentation curves from these studies were used to evaluate the apparent elastic modulus of the probed material using the standard Hertzian model. However, additional information concerning energy dissipation can still be extracted from the same curves to evaluate the viscoelasticity of the material.

Here, we propose a new fitting model to extract the viscoelastic properties of soft materials from AFM force-indentation curves. To construct the explicit relation of force and indentation, we first use a generalization of Maxwell and Kelvin-Voigt models to describe soft materials, and numerically simulate the indentation of such material with a spherical indenter. We show that the proposed Kelvin-Voigt-Maxwell (KVM) model adequately captures the force-indentation curves of materials having different mechanical characteristics. Based on the simulation results, we further propose an explicit force-indentation relation to be fitted to the force-indentation curves. This explicit relation simplifies the association of the mechanical properties with physically meaningful components and processes. Finally, we apply the fitting model to a number of samples, including poroelastic and viscoelastic hydrogels as well as HeLa cells in two different cell cycle phases, interphase and mitotic. We demonstrate that the distinct nature of the hydrogels, arising from the different crosslinking mechanisms, can be described with the fitting model. For the HeLa cells, the mitotic cells had a higher apparent elasticity and a lower apparent viscosity, implying a stiffer actin cortex and a diluted cytoplasm protein concentration, when compared with interphase cells.

Our findings demonstrate that the proposed model can reliably extract viscoelastic properties from conventional force-indentation curves. Moreover, the model is able to assess the contribution of the different elastic and viscous elements, and thus allows a direct comparison between the viscoelastic nature of different materials.

## Results

### The effect of viscoelasticity on force-indentation curves

**Figure 1:**
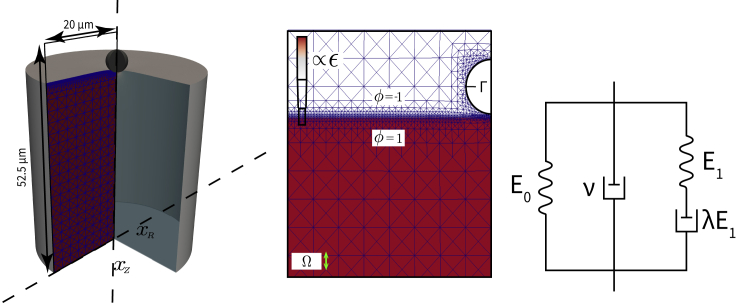
Numerical model for simulating viscoelastic material (Left) Illustration of the 3D scenario with rotational symmetry, and the respective dimensions. The axis of rotation is denoted by $x_{Z}$, the lateral axis by $x_{R}$. (Middle) Grid representation of a 2-dimensional cross section of the domain (Ω). The color code indicates the phase field φ. The red part with ϕ=1 represents the viscoelastic material. The white part with ϕ=-1 represents the liquid medium. Γ denotes the boundary of the indenter, represented by a moving finite-element grid. The right boundary of Ω is the axis of rotation. (Right) Schematic representation of the used neo-Hookean Kelvin-Voigt-Maxwell model

Indenting a biological material with AFM often exhibits a hysteresis between approach and retraction curves which implies the presence of viscoelastic behavior. To account for the viscosity in the quantification of mechanical properties, we developed a numerical model that simulates the process of indenting a viscoelastic half-space immersed in a liquid medium with a rigid spherical indenter (Figure 1). Based on the approach in (Mokbel et al., 2018), the surface of the viscoelastic material is represented by a phase-field \ϕ. Generically, in phase-field models, the surface is not described as sharp, but instead as a diffusive region with a finite thickness (here ε=25 nm). The presence of this diffuse transition region mimics a coarse-grained description of the irregularity of the cell surface and leads to a numerically stable contact algorithm (Mokbel et al., 2018).

In the numerical simulation, the rigid indenter is moved with a prescribed velocity v through the liquid medium toward the viscoelastic material, while the necessary force *F* is constantly computed. The viscosity of the liquid medium is set to the value of water, which gives a negligible force contribution from liquid drag: According to Stokes law, the drag force is $\approx 2.4\cdot 10^{-4}$ nN for a spherical indenter of radius *R* = 2.5 μm at typical velocity of 5 μm/s, which is four orders of magnitude smaller than the typically measured material resistance. Once the indenter comes into contact with the viscoelastic body, the material is deformed and the force *F* starts to increase as viscous and elastic forces counteract the deformation. Once a maximum indentation is reached, the indenter is immediately pulled out of the material in opposite direction.

To determine the viscoelastic properties of the numerical model, we used a combined Kelvin-Voigt-Maxwell model. Such a model can be represented by choosing a combination of springs and dashpots (Figure 1 Right). The involved four material parameters are (*E*_0_) elastic Young's modulus, (η) viscosity, (*E*_1_) an additional Young's modulus which relaxes over time, and (λ) viscoelastic relaxation time. Both elastic components are realized by a hyperelastic neo-Hookean material law.

To validate the numerical model, we first simulated a purely hyperelastic material $\left(\eta =E_{1}=\lambda =0\right)$ using a spring, and compared the resulting force-indentation curves to both the Hertz model (Hertz, 1881) and Ding et al. model (Ding et al., 2017) having the same Young's modulus value as the simulated hyperelastic material:

(Equation 1) $$\begin{array}{ll} F=\frac{4}{3}\frac{E_{0}}{1-\nu^{2}}\sqrt{R\delta^{3}} & \left(\text{Hertz}\;\text{model}\right), \end{array}$$

(Equation 2) $$\begin{array}{ll} F=\frac{4}{3}\frac{E_{0}}{1-\nu^{2}}\sqrt{R\delta^{3}}\left(1-0.15\frac{\delta }{R}\right) & \left(\text{Ding}\;\text{et}\;\text{al}.\right), \end{array}$$

where *F* and *δ* are the force and the indentation depth, respectively, $E_{0},\nu$, and *R* are the Young's Modulus, Poisson's ratio of the material, and radius of the indenter, respectively. While the Hertz model is valid only for very small (linearly) elastic deformations, the model from Ding et al. is applicable for a larger range of deformations as it has been derived numerically using a hyperelastic material law. Both models fitted very well to the simulated data in the range of small indentations and, as expected, the Ding et al. model fitted the data better in the range of large indentations (see Figure S1).

**Figure 2:**
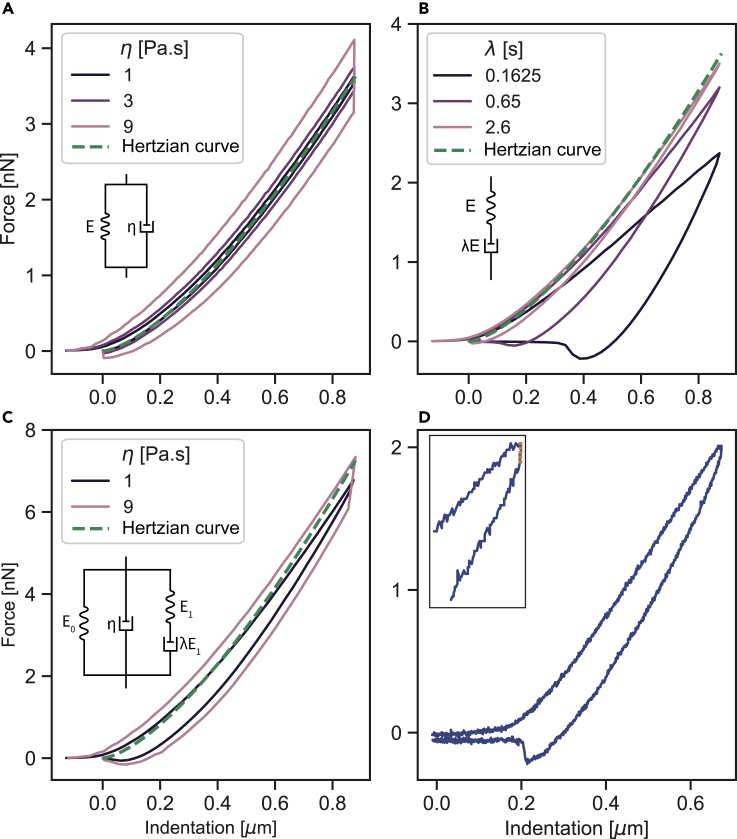
Force-indentation curves of different viscoelastic materials (A) Kelvin-Voigt material simulated with varying viscosity η values and E=1650Pa. The green dashed line is a Hertz model curve with an elasticity of E=1650Pa. (B) Maxwell material simulated with varying relaxation time λ values and E=1650Pa. The green dashed line is a Hertz model curve with an elasticity of E=1650Pa. (C) Kelvin-Voigt-Maxwell (KVM) simulated with varying viscosity η values, λ=0.65s, $E_{0}=1650\;\text{Pa}$, and $E_{1}=1650\;\text{Pa}$. The green dashed line is a Hertz model curve with an elasticity of E=3300Pa. (D) Representative AFM force-indentation curve of a cell. The orange dashed line in the inset highlights the force jump at the end of the approach and beginning of the retraction curves. This jump resembles the jump seen in (A) and (C) for the Kelvin-Voigt and KVM models. For all the subfigures, the radius of the indenter is R = 2.5 μm and the velocity is v = 5 μm/s

To account for viscoelasticity, the simplest two viscoelastic models that can be used are a Kelvin-Voigt solid (spring and dashpot connected in parallel) and a Maxwell liquid (spring and dashpot connected in series). We modeled the indentation of both viscoelastic materials with a spherical indenter (radius *R* = 2.5 μm). In these simulations, the indenter moves into the material at a constant velocity v up to an indentation depth of 0.88 μm, followed by an instantaneous retraction at the velocity −v. Increasing the viscosity of the Kelvin-Voigt material led to a higher deviation from the Hertzian curve Figure 2A. Similarly, the indentation curves deviated more from the Hertzian curves with decreasing the relaxation time of a Maxwell model Figure 2B. It is important to note that when referring, throughout the manuscript, to the mechanical model name such as Kelvin-Voigt or Maxwell model, the intention is the contact model based on that specific mechanical model. Comparing both models, the hysteresis between the approach and retraction curves appeared distinctively different. While the Kelvin-Voigt model shows a hysteresis where the difference between the curves increases with indentation depth, the Maxwell model shows a hysteresis where the difference between the curves is the smallest at the largest indentation depth. In addition, a jump, that is dependent on the viscosity value, can be observed between the approach and retraction curves of the Kelvin-Voigt model. These distinctive features combined together are present when visually inspecting an AFM force-indentation curve (Figure 2D). The Kelvin-Voigt model describes successfully the creep compliance of the material and its ability to regain the original shape after the load is removed. However, it fails in describing the relaxation of a viscoelastic material. On the other hand, the Maxwell model describes very well the stress relaxation but fails in describing the creep of the material (López-Guerra and Solares, 2014). Taken together, to capture both the relaxation and the ability of the indented material to regain its original shape as observed in AFM indentation experiments on soft materials, we modeled the viscoelastic half-space with Kelvin-Voigt (spring $E_{0}$, dashpot η) and Maxwell (spring $E_{1}$, dashpot $\lambda E_{1}$ where λ is the relaxation time) materials connected in parallel (KVM model) (Figure 2C). The force-indentation curves of the KVM model combine the characteristics of the two simple Kelvin-Voigt and Maxwell models. This is specifically observed in the hysteresis as well as the force jump (immediate loss of force) between the approach and retraction curves which resemble very well the representative force-indentation curve of a cell measured by AFM Figure 2D. Also, note that the model captures two characteristics of experimental data which were not reproduced with any other numerical method yet: (i) the onset of a force response already slightly before contact and (ii) the negative force as the indenter detaches from the sample. These contributions stem from diffuse description of the substrate surface and the inclusion of the liquid medium which provides a force before contact (to drain fluid out of the contact region) and a corresponding negative (sinking) force upon detachment.

### Explicit relation of force and indentation

AFM force-indentation curves are typically evaluated by fitting to analytical functions relating the force to the indentation as shown in Equations 1 and 2. In the Ding et al. model, the effects of larger indentation were accounted for by multiplying the Hertz model with an additional term, maintaining, by that, the explicit relation of force and indentation. Here, we followed a similar approach and supplemented the model of Ding et al. with terms that take into account the viscoelastic behavior of the KVM material (Zhang et al., 2017).

The total force acting on the KVM material is equal to the sum of the forces exerted by each of the elements connected in parallel $F_{total}=F_{0}+F_{1}+F_{2};$ where $F_{0}$ is the force exerted by the spring $E_{0}$, $F_{1}$ is the force exerted by the Maxwell element $E_{1}$ and $\lambda E_{1},$ and finally $F_{2}$ is the force exerted by the dashpot η. For elastic solids, the force exerted by the spring $E_{0}$ is linearly related to the value of the elasticity $E_{0}.$ In fact, this can be assumed as the force derived for the Hertz model or any other model that corrects for larger indentations, such as the Ding et al. model. Similarly, the force exerted by the Maxwell element is linearly related to the value of the spring $E_{1},$ however, combined with an exponential decay due to the dashpot $\lambda E_{1}$ dissipation. Finally, the force exerted by the dashpot η can be described as a drag force acting on the segment of the indenter that is in contact with the material. Hence, the force-indentation relation F(δ) is described as follows:

(Equation 3) $$F\left(\delta \right)=F_{elastic}\left(E_{0},\delta \right)\;+\;F_{elastic}\left(E_{1},\delta \right)\cdot \text{exp}\left(\frac{-\alpha_{1}\delta }{\text{v}\lambda }\right)+\alpha_{2}\delta^{\alpha_{3}}R^{\alpha_{4}}\eta \text{v}$$

where,

(Equation 4) $$F_{elastic}\left(E,\delta \right)=\frac{4}{3}\frac{E}{1-\nu^{2}}\sqrt{R\delta^{3}}\left(1-0.15\frac{\delta }{R}\right)$$

and, $E_{0},E_{1},\lambda$, and η correspond to the KVM model elements shown in Figure 2C and v is the velocity of the indenter.

The functional form in Equation 3 contains four fitting parameters, $\left(\alpha_{1},\ldots ,\alpha_{4}\right),$ which are determined from matching Equation 3 to simulated force-indentation curves. Using simulations for a wide range of parameter values, we obtained the values $\alpha_{1}=0.365,\alpha_{2}=7.25,\alpha_{3}=\alpha_{4}=\frac{1}{2}.$ The evolution of the force versus the indentation of the following terms in Equation 3 is shown in Figure S2 and compared to those obtained by the simulations in Figure 2:

As a validation, we used these values with Equation 3 to fit simulated force-indentation curves of different viscoelastic material models such as Kelvin-Voigt, Standard Linear Liquid (SLL), and Kelvin-Voigt-Maxwell model in Figure S3. Using our force-indentation relation, we could determine the viscoelastic characteristic of the material and retrieve the values of the elastic and viscous components. We showed that even different behaviors, such as Kelvin-Voigt and SLL, can be described with the suggested fitting model (See Figure S3).

It is important to note that the fitting model is valid for a specific range of ratios between the Maxwell relaxation time λ and the indentation time $t_{\text{ind}}$ (See Figure S4). If the relaxation time λ is significantly larger than the time in which the material was probed $t_{\text{ind}}$, the effects of $E_{0}$ and $E_{1}$ become redundant and cannot be distinguished anymore. In this case, the material will be depicted as dominantly elastic with an elastic modulus of $E_{0}+E_{1}$. Similarly, if λ is significantly smaller than $t_{\text{ind}}$, the material will relax quickly at the onset of indentation and will be depicted as a pure Kelvin-Voigt model, making $E_{1}$ arbitrary. The fitting range of λ is bound in the algorithm to be dependent on $t_{\text{ind}}$, where the upper and lower limits for the range were set to $10t_{\text{ind}}$ and $\frac{t_{\text{ind}}}{5}$, respectively. These boundaries correspond to a relaxation of $0.9E_{1}$ or $0.006E_{1}$, respectively.

Therefore, in order to give a clear view on the elasticity and the dissipation in different time scales, it is most advantageous to evaluate the total unrelaxed elasticity $E_{\text{u}}=E_{0}+E_{1}$ contained in the material and the apparent elasticity measured in the timescale of the indentation measurement $E_{\text{app}}=E_{0}+E_{1}e^{\frac{-\alpha_{1}\delta }{\text{v}\lambda }}$ as well as the apparent viscosity η. These two moduli lead to a more robust and stable evaluation of the model parameters even at the extreme cases of the ratio $\lambda /t_{\text{ind}}.$

### Viscoelastic properties of hydrogels

**Figure 3:**
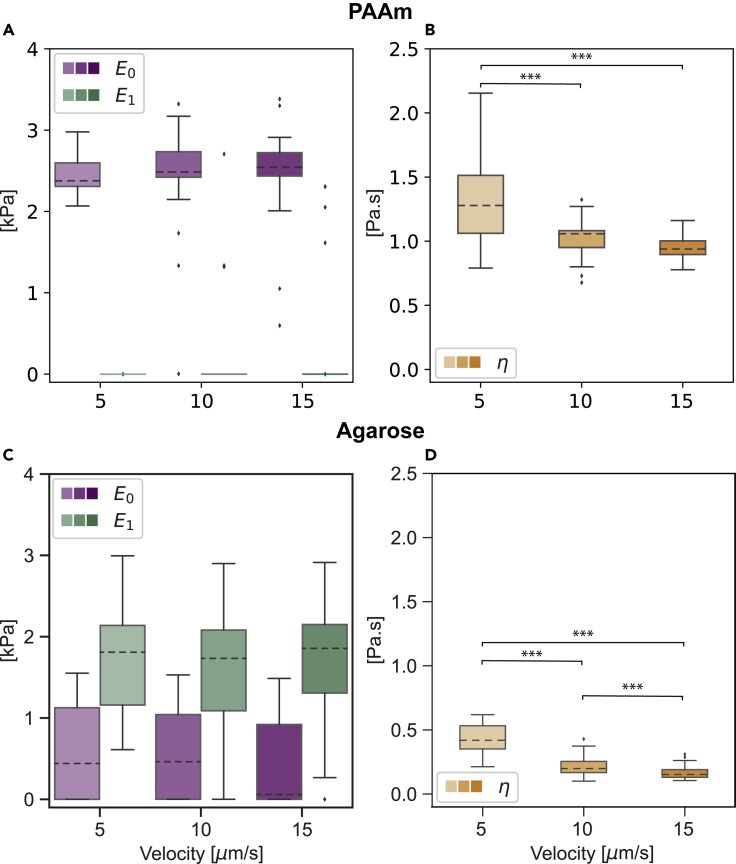
Mechanical properties of hydrogels (A–D) Mechanical characterization of polyacrylamide (PAAm) hydrogel ((A) and (B)) and agarose hydrogel ((C) and (D)) indented with a spherical indenter at different velocities using AFM. (A) and (C) show the elastic moduli $E_{0}$ (purple) and $E_{1}$ (green) for PAAm and agarose hydrogels, respectively. (B) and (D) show the apparent viscosity η (orange) for PAAm and agarose hydrogels, respectively. Data are presented as box-whisker plots (25th, 50th, 75th percentiles, whiskers indicate 10th and 90th percentiles). Results of a statistical test (Kruskal Wallis) are shown. ∗∗∗ denotes p values < 0.001. The number of curves analyzed for every hydrogel at a specific indentation velocity is 27−36. Each hydrogel was measured in four different areas

To investigate the fitting model performance on measured AFM curves, we evaluated the mechanical properties of two hydrogels, polyacrylamide (PAAm) and low-gelling-point agarose hydrogels. The PAAm hydrogel is chemically crosslinked using ammonium persulfate while the agarose hydrogel is physically crosslinked by reducing the temperature of the solution. This difference in the crosslinking mechanisms leads to an inherent difference in their mechanical properties, where the PAAm hydrogel can be described as an elastic network swollen with a liquid solvent (Kalcioglu et al., 2012) and the agarose hydrogel is described as a viscoelastic network. The time dependence of the PAAm hydrogel arises from the necessity of the solvent to move through the elastic polymer network during deformation, while in the agarose hydrogel it comes from the time-dependent nature of the crosslinks between the polymers. We obtained the force-indentation curves of the hydrogels at different piezo velocities 5, 10, and 15 μm/s and evaluated their mechanical properties by fitting Equation 3 to the curves (representative force-indentation curves are shown in Figure S5). Figures 3A and 3C show the two moduli $E_{0}$ and $E_{1}$ for the PAAm and agarose hydrogels, respectively. The apparent viscosity η of the two hydrogels at different velocities is shown in Figure 3B for PAAm and Figure 3D for agarose.

The PAAm hydrogels showed similar values of the Kelvin-Voigt elastic modulus $E_{0}$ at all indentation velocities (2.45±0.04,2.24±0.01,and2.48±0.11kPa), mean ± SE of the mean, for velocities of 5, 10, and 15 μm/s, respectively), while the Maxwell elastic modulus $E_{1}$ was considerably lower for PAAm hydrogels. The agarose hydrogels had higher values of $E_{1}$ compared to $E_{0}$, pointing toward a viscoelastic network that relaxes over time. The apparent viscosity values η for both hydrogels decreased with higher indentation velocities (for PAAm, η=1.3±0.07,1.02±0.03,0.95±0.02 Pa s, and for agarose, η=0.45±0.02,0.21±0.01,0.17±0.01 Pa s for velocities of 5, 10, and 15 μm/s, respectively).

**Figure 4:**
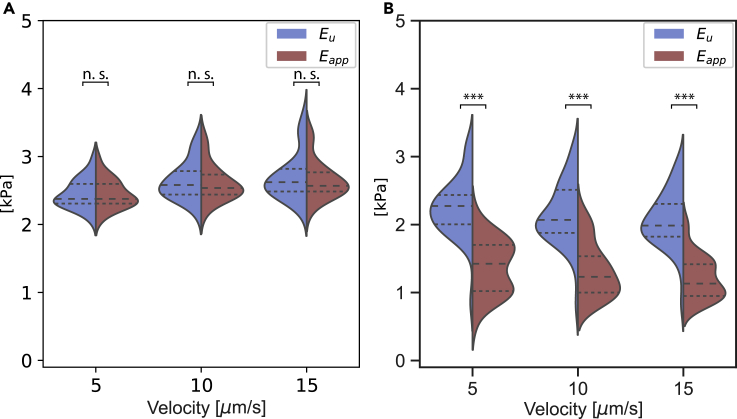
Unrelaxed and apparent Moduli of hydrogels Unrelaxed $E_{\text{u}}$ (blue) and apparent $E_{\text{app}}$ (red) Young's moduli of PAAm (A) and agarose (B) hydrogels quantified at different indentation velocities. Data are presented as violin plots indicating the 25th, 50^th^, and 75th percentiles. Results of a statistical test (Kruskal Wallis) are shown. ∗∗∗ denotes p values < 0.001 and n.s. stands for not significant

The unrelaxed modulus $E_{\text{u}}=E_{0}+E_{1}$ and the apparent modulus $E_{\text{app}}=E_{0}+E_{1}e^{\frac{-\alpha_{1}\delta }{\text{v}\lambda }}$ for PAAm and agarose hydrogels at different indentation velocities are shown in Figures 4A and 4B, respectively. As expected, the PAAm hydrogels had similar values for unrelaxed and apparent moduli ($E_{\text{u}},E_{\text{app}}=2.45\pm 0.04,2.64\pm 0.05,2.7\pm 0.07$ kPa for velocities of 5, 10, and 15 μm/s, respectively), a behavior indicative of the elastic network. The agarose hydrogels, though, showed lower values of the apparent Young's modulus when compared with the unrelaxed modulus ($E_{\text{u}}=2.25\pm 0.07,2.15\pm 0.08,2.07\pm 0.06$ kPa and $E_{\text{app}}=1.38\pm 0.06,1.33\pm 0.06,1.21\pm 0.05$ kPa for velocities of 5, 10, and 15 μm/s, respectively). This difference in the values of the unrelaxed and the apparent moduli originates from the relaxation of the viscoelastic network during the measurement time. Here, we demonstrated the ability of the model to describe the mechanical behavior of two different hydrogels.

### Viscoelastic properties of HeLa cells in interphase and mitosis

**Figure 5:**
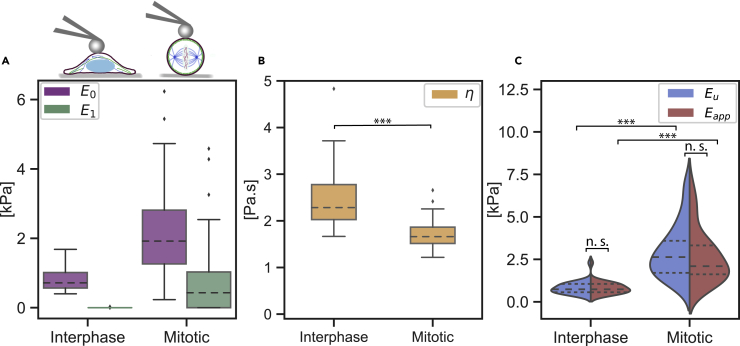
Mechanical properties of interphase and mitotic HeLa cells (A) The elastic moduli $E_{0}$ (purple) and $E_{1}$ (green). (B) The apparent viscosity η (yellow). Data in (A) and (B) are presented as box-whisker plots (25th, 50th, 75th percentiles, whiskers indicate 10th and 90th percentiles). Results of a statistical test (Kruskal Wallis) are shown. ∗∗∗ denotes p values < 0.001. (C) The unrelaxed $E_{\text{u}}$ (blue) and apparent $E_{\text{app}}$ (red) moduli of cells in both cell cycle phases. Data are presented as violin plots indicating the 25th, 50th and 75th percentiles. Results of a statistical test (Krus,kal Wallis) are shown. ∗∗∗ denotes p values < 0.001 and n.s. stands for not significant. For every cell cycle phase two independent experiments were performed and a total of 32 cells were measured

We measured the viscoelastic properties of interphase and mitotic HeLa cells (representative force-indentation curves are shown in Figure S6). Comparing the Kelvin-Voigt modulus $E_{0}$, the interphase cells appeared overall more compliant than the mitotic cells ($E_{0}=0.8\pm 0.06$ kPa for interphase and $E_{0}=2.2\pm 0.3$ kPa for mitotic) as shown in Figure 5A. Interestingly, the mitotic cells also had a significantly higher value of the Maxwell modulus $E_{1}$ which relaxes during the indentation measurement time ($E_{1}=0.0\pm 0.0$ kPa for interphase and $E_{1}=0.76\pm 0.17$ kPa for mitotic). The relaxing elastic component could be attributed to the difference in the actin cortex arrangement between spread interphase cells and round mitotic cells. Furthermore, the apparent viscosity η was higher for the interphase cells (η=2.71±0.18 Pa.s for interphase and η=1.71±0.06 Pa.s for mitotic cells).

Owing to the presence of a Maxwell modulus component $E_{1}$ in the mechanical characterization of the mitotic cells, their apparent Young's Modulus appeared, although not statistically significant, lower when compared with the unrelaxed modulus ($E_{\text{u}}=3.0\pm 0.28$ kPa and $E_{\text{app}}=2.7\pm 0.28$ kPa) as shown in Figure 5C. The interphase cells had similar values of the unrleaxed ($E_{\text{u}}=0.8\pm 0.06$ kPa) and apparent ($E_{\text{app}}=0.8\pm 0.07$ kPa) moduli. Here, we showed that the viscoelastic properties of mitotic and interphase cell states can now be easily compared via a simple force-indentation measurement.

## Discussion

In the last few years, AFM became the gold standard method for probing the rheological properties of small-sized soft materials that, otherwise, could not be probed with conventional viscometers and rheometers. For adequate characterization of the materials' viscoelastic behavior with AFM, one should observe the response of the material in time or frequency domains. Time-dependent static measurements are usually applied via step-hold experiments where the force relaxation or the material creep are recorded. An analytical model is then fitted to the recorded force or indentation signals extracting the viscoelastic properties (Darling et al., 2007, 2008; Moreno-Flores et al., 2010). Alternatively, dynamic measurements can be applied by moving the cantilever in a sinusoidal manner and recording the amplitude and the phase shift of the cantilever deflection. The complex modulus of the material is then evaluated at the different oscillation frequencies of the cantilever (Alcaraz et al., 2003; Rother et al., 2014). Both methods, step-hold and sinusoidal oscillation, require the user to prepare elaborate acquisition and analysis processes with lengthy measurement times.

Force-indentation measurements, where the cantilever is linearly lowered to indent the sample and then retracted back, are still one of the most used methods for probing soft materials with AFM. A Hertzian model is conventionally used to extract the elastic modulus of the probed sample from the force-indentation curves. The hysteresis present between the load and unload curves of the sample confirms that the Hertzian contact mechanics assumption of a purely elastic material is not met. Thus, a few studies have attempted to derive the viscoelastic properties of the materials from the hysteresis between the approach and retraction curves (Rebelo et al., 2013; Nawaz et al., 2012; Mathur et al., 2001). For example, Rabelo et al. (Rebelo et al., 2013) estimated the viscoelastic properties of human kidney epithelial cancer cells from force-indentation curves. The elastic modulus was obtained by fitting the Hertz model, while the apparent viscosity was calculated from the hysteresis between approach and retraction curves of the cantilever. More specifically, the work lost in the approach-retraction cycle was associated with the internal friction forces of the sample. In this work, the calculation of the apparent viscosity required, among other steps, the integration of the approach-retraction curves to calculate the slopes over the indentation range. Nonetheless, this method relies on estimating the slope of noisy data and it assumes an elastic fit for the indentation and retraction curves.

Other recent studies have suggested fitting both the approach and retraction parts of the force curve with a model that accounts for the viscoelastic dissipation (Brückner et al., 2017; De Sousa et al., 2017; Efremov et al., 2017; Garcia et al., 2020). The development of the models relied on the viscoelastic contact mechanics derivation by Lee and Radok (Lee and Radok, 1960) and the application of Ting's model (Ting, 1966) for extending the solution to the retraction part of the curve. In each of these studies, a mechanical model, such as a standard linear solid (SLS) or power law rheology, is chosen to describe the material's mechanical nature. Efremov et al. (Efremov et al., 2017) suggested using an SLS model for describing hydrogels and power law rheology for describing cells. The necessity to alternate between models originated from the difference in the behavior of cells and hydrogels in the extreme cases of t→0 and t→∞. In these cases, the SLS model behaves as a purely elastic material, where the elastic modulus $E_{0},$ having higher values at short times, relaxes to another constant, yet lower, elastic modulus value at longer times $E_{\infty }.$ The power law model, on the other hand, shows a monotonic decrease of the elastic modulus value over time. In these approaches, the user is required to select a model, SLS or power law, even though the material's mechanical properties is yet to be determined. In addition, the suggested fitting models do not give an explicit relation between the force and indentation signals, and require a rather iterative process of solving integrals numerically. Lastly, the retraction part of the curve includes adhesion-related artifacts which hinder the accurate fitting of the viscoelastic model.

Here, we developed a new model for fitting the AFM force-indentation curves and extracting the viscoelastic behavior of the material. We showed that combining the simplest two viscoelastic mechanical models, Kelvin-Voigt and Maxwell, will account for the hysteresis as well as the regaining of the initial material shape. Our numerical method uses fundamental physical laws to predict the force-indentation curve for given viscoelastic parameters. Still, our goal is to use directly the measured force-indentation curve to predict the viscoelastic parameters. As the numerical model cannot be easily inverted, we use the numerical results for a broad range of parameters to extract the functional dependence by means of a fitting routine.

Thus, to extract the viscoelastic properties from AFM force-indentation curves, we proposed a fitting model that describes an explicit relation between the force and the indentation signals in the approach curve. Unlike the three mechanical component models, such as SLS, the Kelvin-Voigt-Maxwell (KVM) can still describe the viscosity of the materials at very short times. In the force-indentation curves, this is translated to the presence of hysteresis between the approach and retraction parts at low and high indentation velocities, a characteristic feature of force-indentation curves of cells (Efremov et al., 2017). In addition, due to the flexibility of the model to describe simpler mechanical behaviors, such as Kelvin-Voigt or SLL (see Figure S3), it is suitable to be fitted to force-indentation curves of hydrogels that exhibit no hysteresis at low indentation velocities (Efremov et al., 2017). Ultimately, using our fitting method, the user is not required to alternate between different models to capture the mechanical nature of different materials and can conveniently compare their fitted parameters.

We used PAAm and agarose hydrogels, each having a different crosslinking mechanism, to validate the fitting model. The PAAm gels exhibited, over the range of different indentation velocities, an elastic behavior accompanied with an apparent viscosity that probably arises from the liquid solvent moving within the elastic polymer network. On the other hand, the agarose gels displayed a viscoelastic behavior that can be explained by the physical crosslinking mechanism of such gels which allows the rearrangement of the polymer network and its contact points during the indentation process.

Finally, we indented HeLa cells in interphase and mitosis. The interphase cells seemed overall more compliant. However, the mitotic cells exhibited higher values of the Maxwell modulus $E_{1}$ that lead to higher viscoelastic dissipation during the indentation. Additionally, the apparent viscosity was higher for the interphase cells. These mechanical differences between the cell cycle phases were also highlighted in a number of other studies (Fischer-Friedrich et al., 2014, 2016; Taubenberger et al., 2020). The higher overall stiffness of the mitotic cells was repeatedly attributed to the stiffer actin cortex of these cells which plays an important role in the rounding and the division processes. On the other hand, the lower apparent viscosity of mitotic cells was supposed to be an outcome of the cytoplasmic protein dilution due to water entry and the cell volume increase at this phase (Mchedlishvili et al., 2018; Taubenberger et al., 2020). Still, in the current fitting model, the effect of the stiff substrate on the force was neglected. However, a calculation of the over-estimation in the force values for HeLa cells is provided in the AFM analysis section. For future work, one might include a correction factor to the model that is dependent on the material thickness and the indentation magnitude, similarly to Niu et al. (Niu and Cao, 2014), using the numerical model that we developed for this study.

In summary, here we developed an analysis method to extract the viscoelastic properties of soft materials from AFM force-indentation curves. We demonstrated the applicability of our method, using the same mechanical model to both hydrogels and cells. This method can be easily incorporated into the conventional analysis of the force-indentation curves to broaden the characterization of the rheological properties of soft materials and specifically biological matter.

### Limitations of the study

The fitting model proposed in this study quantifies the viscoelastic properties of materials probed with AFM from the same force-indentation curves generated for conventional Hertzian fitting. The current fitting model neglects the effect of the stiff substrate underlying the probed material. In the future, a correction factor can be included that takes into account the probed material thickness and the indentation magnitude. One additional limitation to the fitting model is the contact point estimation. In this study, we fitted a line and a polynomial function only for the initial estimation of the contact point, which was then fitted again using the force-indentation relation presented in Equation 3. Defining the correct contact point, specifically for cells and tissue, has been a challenge for the majority of AFM indentation applications. Few studies suggested the use of multilayered models that take into account the indentation of a pericellular layer (Sokolov et al., 2007; Rusaczonek et al., 2019). These approaches can be combined with our model for a more precise evaluation of the viscoelastic properties of cells.

## STAR★Methods

### Key resources table

**Table:** 

REAGENT or RESOURCE	SOURCE	IDENTIFIER
**Chemicals, peptides, and recombinant proteins**		

Acrylamide	Sigma-Aldrich	Cat# A8887-100G
N,N′-Methylenebisacrylamide	Sigma-Aldrich	Cat# 146072-100G
N,N,N′,N′-Tetramethylethylendiamin	Sigma-Aldrich	Cat# 411019-100ML
low-gelling-point agarose	Sigma-Aldrich	Cat# A0701-100G
Triethylamine	Sigma-Aldrich	Cat# T0886-100ML
Glutaraldehyde	Sigma-Aldrich	Cat# G6257-100ML
ammonium persulphate	Sigma-Aldrich	Cat# GE17-1311-01
DMEM, high Glucose	Thermo Fisher Scientific	Cat# 11965092
Fetal bovine serum	Sigma-Aldrich	Cat# F7524-500ML

**Deposited data**		

Raw AFM force-indentation data	This Paper, Mendeley data	https://doi.org/10.17632/c2gccnfkgd.2
Numerical model source code	This Paper, Mendeley data	https://doi.org/10.17632/c2gccnfkgd.2
Fitting model code	This Paper, Mendeley data	https://doi.org/10.17632/c2gccnfkgd.2

**Experimental models: Cell lines**		

HeLa Kyoto EGFP-alpha-tubulin/H2B-mCherry	Provided by the laboratory of Mathieu Piel, originally obtained as explained in (Steigemann et al., 2009)	RRID: CVCL_L802

**Software and algorithms**		

Python	https://www.python.org/downloads/	Version 3.7
PyJibe	https://github.com/afm-analysis/pyjibe	version 0.13.2

**Other**		

PNP-TR-TL cantilevers	NanoWorld	PNP-TR-TL-50
polystyrene beads	Microparticles GmbH	PS-R-5.0
13 mm	Marienfeld	Cat# 0111530

### Resource availability

#### Lead contact

Further information and requests for resources should be directed to and will be fulfilled by the lead contact, Shada Abuhattum (shada.abuhattum@mpl.mpg.de)

#### Materials availability

The study did not generate any materials.

### Experimental model and subject details

The cell line used in this study were developed from HeLa cell line (originally derived from female tissue) provided by Matthieu Piel's lab and originally obtained from (Steigemann et al., 2009). The cells were cultured at 37°C and 5% CO2 in high glucose DMEM (Dulbecco's Modified Eagle Medium) supplemented with 10% foetal bovine serum (FBS). Cells were not authenticated in this work.

### Methods details

#### The numerical model

The numerical method is based on the phase-field model of contact and fluid–structure interaction problems presented in (Mokbel et al., 2018). This previous work describes an efficient method to simulate the interaction between a liquid and a (visco-) elastic phase. A monolithic Navier-Stokes equation was solved for the velocity field in both, the fluid and the elastic domain. Viscous and elastic forces were restricted to the respective domains via multiplication with characteristic functions. The elastic stress was obtained by an additional Oldroyd-B-like equation which was coupled to the Navier-Stokes equation.

In the present work we adopt these fundamental features and extend the model in order to simulate a three-phase scenario, including the interaction between the viscoelastic substrate and the surrounding liquid medium together with the rigid spherical indenter. The main modification with respect to (Mokbel et al., 2018) is the inclusion of the rigid indenter, which is detailed below.

As in (Mokbel et al., 2018), we consider a rotationally symmetrical setup and thus efficiently simulate a 3D scenario on a 2D grid (see Figure 1). Let us note here that we omit the surface tension between liquid and substrate phase in this work. The model comes with a full Eulerian description of the (common) velocity field $v:\text{Ω}\to R^{n}$ in the whole computational domain $\text{Ω}\subset R^{n}.$ We denote by $v:=\left(v_{Z},v_{R}\right)$ the lateral and the normal component, referring to the axis of symmetry. Furthermore we introduce by $\left(x_{z},x_{r}\right)$ the lateral and the normal component of the position of grid points. We set $v_{R}=0,x_{r}=0$ on the axis of symmetry and $v_{Z}=0$, $x_{z}=0$ at the bottom boundary (i.e. at the bottom of the substrate). Since it is not necessary to solve any hydrodynamics inside the indenter, we simply represent the indenter phase by a spherical hole in the computational domain Ω (see Figure 1). On the indenter boundary Γ⊂∂Ω we set $v_{Z}=v$ and $v_{R}=0$, where v is the prescribed (constant) indenter velocity introduced in section e.

The motion of the indenter is realized by a moving Finite-Element grid with velocity $v_{G}:\text{Ω}\to R$ which describes the movement of grid points in lateral direction. We choose a concertina-like movement, $v_{G}=\text{v}\cdot \text{min}\left(1,x_{z}/x_{I}\right)$, where $x_{I}$ denotes the lateral position of the advancing (bottom) point of the indenter. An ALE approach is used to account for the moving grid. This amounts to replacing material derivatives by $\partial_{t}+\left(v-{\left(v_{G},0\right)}^{T}\right)\cdot \nabla$, where $\partial_{t}$ is the time derivative along a moving grid point.

The indenter boundary experiences a force which is calculated by $2\pi \underset{\text{Γ}}{\int }S\cdot n$, where $S=-pI+\nu \left(\nabla v+\nabla v^{T}\right)$ is the stress tensor, n is the normal on Γ pointing inside Ω, p the pressure and ν the viscosity of the region in contact with the indenter. The lateral component of this force yields the viscoelastic response of the material, denoted by *F*. In summary, the rigid indenter as a third phase in our model extension is simply modeled as a semicircular part Γ⊂∂Ω of the domain boundary, which interacts with the remaining phases by means of a velocity boundary condition. The force *F* is calculated at the end of each simulation time step from the current values for v and p. Let us further note that in this work we avoid adhesion between the indenter and the elastic substrate by setting a phase field boundary condition (here φ=−1 on Γ, see Figure 1).

In the previous work, the structure could either be modeled as purely elastic, or as a Kelvin-Voigt or Maxwell material. The choice of the material was made on the basis of certain model parameters (see Mokbel et al. (2018)). Since we consider a combined KVM material in the present work, a Kelvin-Voigt and a Maxwell stress contribute to the total stress in parallel. We now solve two Oldroyd-B-like equations and set the model parameters once according to Kelvin-Voigt and once according to Maxwell. Consequently we obtain two different quantities that add up to the total elastic stress. The system now includes two strain tensors $\sigma_{0}$, $\sigma_{1}$. To describe their evolution we adopt the notation from (Mokbel et al., 2018) in the following. The corresponding elastic stresses are $\frac{1}{3}E_{0}\left(\varphi \right)\left(\sigma_{0}-I\right)$ and $\frac{1}{3}E_{1}\left(\varphi \right)\left(\sigma_{1}-I\right)$ in order to differentiate between Kelvin-Voigt and Maxwell elasticity. Note, that the elastic parameter is a field dependent on the phase field φ. In the ambient fluid $E_{0}=E_{1}=0$ applies. The strain tensors are calculated by

$$\lambda_{i}\left(\varphi \right)\left(\partial^{\cdot }\sigma_{i}-\nabla v^{T}\cdot \sigma_{i}-\sigma_{i}\cdot \nabla v\right)+\alpha_{i}\left(\varphi \right)\left(\sigma_{i}-I\right)=0\;\text{for }i=0,1.$$

In the viscoelastic material we chose $\alpha_{0}=0$, $\alpha_{1}=1$, $\lambda_{0}=1$ and $\lambda_{1}=\lambda$, where the latter value is identical to the Maxwell relaxation time introduced in this work. In the ambient fluid we chose $\alpha_{0}=\alpha_{1}=1$, $\lambda_{0}=\lambda_{1}=0$. Furthermore, we defined a constant mobility m=1e−11 m^3^s/kg, an interface width ε=2.5e−8 m and a fixed grid size of h=2.5e−6 m away from the interface. At the interface the grid size is h=7.8125e−8 m. The time step size is τ=2.5e−5 s.

The numerical simulation method was implemented in the Finite-Element toolbox AMDiS (Vey and Voigt, 2007; Witkowski et al., 2015) which can be downloaded from (https://gitlab.math.tu-dresden.de/iwr/amdis). Please note that AMDiS requires the Linux operating system and many further external libraries to be installed. Detailed installation instructions can be found under the above link. Our numerical code for the present simulations can be obtained at Mendeley data DOI: 10.17632/c2gccnfkgd.2 and needs to be linked to AMDiS. A tutorial in the AMDiS folder doc → tutorial provides further help and describes how codes can be compiled and linked to AMDiS. Additionally, we provide an exemplary run script. Output files are created in *.vtk* or *.pvd* format, which can be opened e.g. with Paraview (https://www.paraview.org/). The file names for phase field data are denoted beginning with ”ch”. Moreover, a file named ”output” is produced in the simulation, which contains real time, force and indentation data in the first, second and third column, respectively.

#### Hydrogel preparation

To prepare the hydrogels for AFM indentation measurements, stock solutions were initially prepared as follows: A stock solution of PAAm hydrogel was prepared by mixing the following components in PBS to reach the indicated final percentages: 7.5% acrylamide (Sigma-Aldrich, Germany), 0.06% N,N′-Methylenebisacrylamide (Bis-acrylamide) (Sigma-Aldrich, Germany) and 0.3% N,N,N′,N′- Tetramethylethylenediamine (TEMED) (Sigma-Aldrich, Germany). The stock solution of agarose hydrogels was prepared by dissolving low-gelling-point agarose (Sigma-Aldrich, Germany) in warmed ddH_2_O to a final concentration of 0.5%. The preparation of hydrogel samples for AFM indentation measurements was done according to a previously described method (Moshayedi et al., 2010). Briefly, round glass cover slips (13mm∅, Marienfeld) were first washed with 1 N NaOH for 30 min then with ddH_2_O, 100% ethanol and ddH_2_O. After drying, 0.1% v/v of chloroform (Sigma-Aldrich, Germany) mixed with 0.1% v/v of triethylamine (Sigma-Aldrich, Germany) was added to the cover slips for another 30 min and then washed with ddH_2_O. Finally, a solution of 0.5% glutaraldehyde (Sigma-Aldrich, Germany) in ddH_2_O was added to the cover slips for 30 min followed by a washing step with ddH_2_O. To polymerise the PAAm solution, ammonium persulphate (Sigma-Aldrich, Germany) was added with a final concentration of 1%. 35 μL of each hydrogel stock solution were added onto ethanol cleaned foil, then the glass cover slip was placed on top of the solution. After 30 min at room temperature the PAAm were fully polymerised and the agarose gels were crosslinked. Then the hydrogels were kept in PBS for at least 24 hours for stabilization before the AFM indentation measurements.

#### HeLa cell culture

HeLa Kyoto EGFP-alpha-tubulin/H2B-mCherry (RRID: CVCL-L802) cells, kindly provided by Matthieu Piel (Institut Curie), originally derived in (Steigemann et al., 2009) (transfected with pH2B-mRFP and pmEGFP-α-tubulin-IRES-puro2b plasmids), were cultured at 37°C and 5% CO2 in high glucose DMEM (Dulbecco's Modified Eagle Medium, 11965092) supplemented with 10% foetal bovine serum (FBS) and 10 units/mL penicillin /streptomycin. Cells were subcultured at approximately 70% confluency. Briefly, the cell culture medium was aspirated, cells were washed with PBS and incubated with 0.25% Trypsin/EDTA for 3–5 min at 37°C. Detached cells were resuspended in culture medium, centrifuged at 200 × g for 3 min and counted using a haemocytometer. Cells were seeded 24 or 48 hours prior to the experiments, at a density of 20,000 or 10,000 cells/cm^2^ in 35 mm tissue culture plates (TTP, Switzerland, 93040).

#### AFM measurements

AFM measurements were preformed using a Nanowizard 4 (JPK BioAFM, Bruker Nano GmbH, Berlin). To indent the samples, PNP-TR-TL (Nanoworld) cantilevers with a nominal spring constant of 0.08 mN/m were modified by gluing 5 μm diameter polystyrene beads (microparticles GmbH, Berlin) to the underside of the cantilevers using two component glue (Araldite Rapid, Huntsman Advanced Materials, Switzerland). The cantilevers were calibrated prior to each experiment using the thermal noise method (Hutter and Bechhoefer, 1993) and their accurate spring constant ranged between 0.047-0.059 mN/m. For PAAm and agarose hydrogels, the cantilever was lowered with a constant velocity (5, 10, or 15 μm/s) toward the surface of the sample until a force of 2 nN for agarose and 4 nN for PAAm was reached. These force set points accounted for an indentation in the range of 0.5–1 μm. For HeLa cells, the cantilever was lowered with a constant velocity of 2 μm/s and the cells were indented until a force of 2 nN was reached, which accounted for an indentation depth in the range of 0.5–1.5 μm.

#### AFM force-indentation analysis

For analysis and implementation of the fitting model described in Equation 3 we used the freely available, open-source analysis software PyJibe (Müller, 2019). Our fitting model is available as a PyJibe extension (the fitting algorithm and the instruction of use are uploaded to Mendeley data). For every force-indentation curve, a correction for the tip-sample separation was performed. Then, an initial contact point was calculated. The Python package nanite offers several different methods for the initial estimation (Müller et al., 2019). We found that the most suitable method for our curves is to fit a line and a polynomial to the baseline and the indentation parts of the force *F* as described below (https://nanite.readthedocs.io/en/stable/sec_code_reference.html#nanite.poc.poc_fit_line_polynomial):The linear baseline (*δ* < 0) is modeled as

F=mδ+d

with the fit parameters slope of the linear baseline *m* and force-offset *d*. The indentation part (*δ* > 0) is modeled as

$$F=\frac{\delta^{3}}{a\delta^{2}+b\delta +c}+m\delta +d$$

with the polynomial fit parameters *a*, *b*, and *c*. For small indentations, this function exhibits a linear and only slightly cubic behavior:

$$y\approx \delta^{3}/c+m\delta +d$$

And for large indentations, this function is linear:

$$y\approx \left(\frac{1}{a}+m\right)\delta$$

The point of contact is defined as δ=0. This estimation is used as an initial guess for the fitting model, where the contact point is fitted again.

Finally, the curve was fitted with the model fit in Equation 3. We used Nelder-Mead minimization method from the lmfit Python package as it appears more stable for fitting of exponents than Levenberg-Marquardt (the default in PyJibe). A Poisson's ratio of 0.5 was assumed.Due to the spherical shape of the mitotic HeLa cells an effective radius $R_{\text{eff}}$ was calculated before performing the fit:

$$\frac{1}{R_{\text{eff}}}=\frac{1}{R_{\text{cell}}}+\frac{1}{R_{\text{bead}}}$$

where $R_{\text{cell}}$ is the averaged radius of the mitotic cells and $R_{bead}$ is the radius of the spherical indenter. An additional correction was applied to the force-indentation curves of round mitotic HeLa cells that arises from the additional stress that the cells encounter from the bottom of the plate, which causes an additional deformation of the spherical shape. These corrections have been described in detail in (Dokukin et al., 2013; Glaubitz et al., 2014) and require the following alteration to the indentation signal prior to performing the fit:

$$\delta_{corrected}=\delta_{measured}\text{∗}K$$

$$K=\frac{R_{\text{cell}}^{1/3}}{R_{\text{cell}}^{1/3}+R_{\text{eff}}^{1/3}}$$

where δ denotes the indentation signal and *K* is the correction factor.For our analysis $R_{\text{cell}}$ = 7.5 μm, $R_{\text{bead}}$ = 2.5 μm, $R_{\text{eff}}$ = 1.875 μm and *K* = 0.613.

Our model is developed for the general case without taking into account the effect of the stiff surface on the fitted parameters. Indenting cells with a large spherical indenter is often affected by the underlying stiff surface (Dimitriadis et al., 2002; Garcia, 2020; Garcia et al., 2020). Garcia et al. (Garcia and Garcia, 2018) proposed a theory taking into account the finite viscoelastic layer to calculate the force *F* as a function of indentation *I*:

(Equation 5) $$F\left(I\right)=\overset{N}{\underset{j=0}{\sum }}\alpha_{j}\int_{0}^{t}\varphi_{\text{KVM}}\left(t-t^{\prime }\right)\frac{d}{dt^{\prime }}\left(I{\left(t^{\prime }\right)}^{\beta^{j}}\right)dt^{\prime }$$

where coefficients $\alpha_{j}$ and $\beta_{j}$ depend on the geometry and the thickness of the layer, the relaxation function $\varphi_{\text{KVM}}=E_{0}+E_{1}e^{-t/\lambda }+\eta \delta \left(t\right)$ and δ(t) is the delta of Dirac. Solving the integral in Equation 3 for the general case is challenging and knowledge regarding I(t) needs to be available (see solution for SLS model in (Niu and Cao, 2014)). Still, in our study we calculate the final apparent Young's modulus $E_{\text{app}}$ and apparent viscosity η which can substitute the relaxation function by a simpler one $\varphi =E_{\text{app}}+\eta \delta \left(t\right)$ as described in (Garcia and Garcia, 2018) to estimate the error of neglecting the bottom-effect, where the solution for the force-indentation for the first two indexes j=0,1 is as follows (Garcia and Garcia, 2018):

$$F\left(I\right)=\frac{16}{9}\sqrt{RI}\left[\frac{9}{2}\eta \dot{I}+E_{\text{app}}I\right]+1.133\frac{16}{9h}RI\left[6\eta \dot{I}+E_{\text{app}}I\right]+O\left(\frac{I^{\frac{3}{2}}}{h^{2}}\right)$$

where *R* is the radius of the tip, *h* is the thickness of the sample.

The second term allows us to calculate the additional force resulting from the rigid substrate and is dependent on the thickness of the sample and the tip dimension.

For the HeLa cells, substituting the values indicated in Figure S6 as well as R = 2.5 μm, $h_{\text{interphase}}$ = 5 μm as described in (Sanchez et al., 2021), $h_{\text{mitotic}}$ = 15 μm and $\dot{I}=\text{v}$ = 2 μm/s leads to:

$$F_{\text{interphase}}\left(I\right)=\underset{2\text{nN}}{\underset{︸}{\frac{16}{9}\sqrt{RI}\left[\frac{9}{2}\eta \dot{I}+E_{\text{app}}I\right]}}+\underset{0.6\text{nN}}{\underset{︸}{1.133\frac{16}{9h}RI\left[6\eta \dot{I}+E_{\text{app}}I\right]}}$$

$$F_{\text{mitotic}}\left(I\right)=\underset{2.5\text{nN}}{\underset{︸}{\frac{16}{9}\sqrt{RI}\left[\frac{9}{2}\eta \dot{I}+E_{\text{app}}I\right]}}+\underset{0.2\text{nN}}{\underset{︸}{1.133\frac{16}{9h}RI\left[6\eta \dot{I}+E_{\text{app}}I\right]}}$$

Neglecting the bottom-effect leads to over estimation of the force that is larger for the interface cells (by 30%) than the mitotic cells (by 8%). The bottom-effect can be reduced by choosing a sharper tip (e.g. conical) for measurements of spread cells.

### Quantification and statistical analysis

#### Statistical details

• The data in Figures 3, 5A, and 5B are presented as box-whisker plots (25th, 50th, 75th percentiles, whiskers indicate 10th and 90th percentiles).
• The data in Figures 4 and 5C are presented as violin plots indicating the 25th, 50th and 75th percentiles.

The values in the manuscript are reported as mean ± standard error of the mean. For the hydrogels, every point used in the plots represents one force-indentation curve. For the cells, every point represents one cell measurement and is the median value of three force-indentation curves performed for every cell. The number of the points is indicated in the caption of the figures.

#### Statistical analysis

The statistical analysis were performed using Kruskal Wallis to determine the differences among multiple groups, followed by t-test post hoc analysis to obtain p values. In all figures, p value significance is indicated as follows: ∗∗∗, p < 0.001; ns (not significant), p >0.05. All tests were performed using python scipy statistical package (Virtanen et al., 2020).

## Data Availability

• All AFM force-indentation curves data have been deposited at Mendeley data, and are publicly available as of the date of publication, DOI is listed in key resources table. • All original numerical and fitting model code have been deposited at Mendeley data, and are publicly available as of the date of publication, DOI is listed in key resources table. • Any additional information required to reanalyze the data reported in this paper is available from the lead contact upon request.

## References

[bib1] Abuhattum S., Kim K., Franzmann T.M., Eßlinger A., Midtvedt D., Schlüßler R., Möllmert S., Kuan H.S., Alberti S., Zaburdaev V. (2018). Intracellular mass density increase is accompanying but not sufficient for stiffening and growth arrest of yeast cells. Front. Phys..

[bib2] Alcaraz J., Buscemi L., Grabulosa M., Trepat X., Fabry B., Farré R., Navajas D. (2003). Microrheology of human lung epithelial cells measured by atomic force microscopy. Biophys. J..

[bib3] Benaglia S., Amo C.A., Garcia R. (2019). Fast, quantitative and high resolution mapping of viscoelastic properties with bimodal afm. Nanoscale.

[bib4] Brückner B.R., Nöding H., Janshoff A. (2017). Viscoelastic properties of confluent mdck ii cells obtained from force cycle experiments. Biophys. J..

[bib5] Darling E.M., Topel M., Zauscher S., Vail T.P., Guilak F. (2008). Viscoelastic properties of human mesenchymally-derived stem cells and primary osteoblasts, chondrocytes, and adipocytes. J. Biomech..

[bib6] Darling E.M., Zauscher S., Block J.A., Guilak F. (2007). A thin-layer model for viscoelastic, stress-relaxation testing of cells using atomic force microscopy: do cell properties reflect metastatic potential?. Biophys. J..

[bib7] De Sousa J.S., Santos J.A.C., Barros E.B., Alencar L.M.R., Cruz W.T., Ramos M.V., Mendes Filho J. (2017). Analytical model of atomic-force-microscopy force curves in viscoelastic materials exhibiting power law relaxation. J. Appl. Phys..

[bib8] Dimitriadis E.K., Horkay F., Maresca J., Kachar B., Chadwick R.S. (2002). Determination of elastic moduli of thin layers of soft material using the atomic force microscope. Biophys. J..

[bib9] Ding Y., Xu G.K., Wang G.F. (2017). On the determination of elastic moduli of cells by AFM based indentation. Sci. Rep..

[bib10] Diz-Muñoz A., Fletcher D.A., Weiner O.D. (2013). Use the force: membrane tension as an organizer of cell shape and motility. Trends Cell Biol..

[bib11] Dokukin M.E., Guz N.V., Sokolov I. (2013). Quantitative study of the elastic modulus of loosely attached cells in afm indentation experiments. Biophys. J..

[bib12] Efremov Y.M., Wang W.H., Hardy S.D., Geahlen R.L., Raman A. (2017). Measuring nanoscale viscoelastic parameters of cells directly from afm force-displacement curves. Sci. Rep..

[bib13] Escolano J.C., Taubenberger A.V., Abuhattum S., Schweitzer C., Farrukh A., Del Campo A., Bryant C.E., Guck J. (2021). Compliant substrates enhance macrophage cytokine release and nlrp3 inflammasome formation during their pro-inflammatory response. Front. Cell Dev. Biol..

[bib14] Fabry B., Maksym G.N., Butler J.P., Glogauer M., Navajas D., Fredberg J.J. (2001). Scaling the microrheology of living cells. Phys. Rev. Lett..

[bib15] Fischer-Friedrich E., Hyman A.A., Jülicher F., Müller D.J., Helenius J. (2014). Quantification of surface tension and internal pressure generated by single mitotic cells. Sci. Rep..

[bib16] Fischer-Friedrich E., Toyoda Y., Cattin C.J., Müller D.J., Hyman A.A., Jülicher F. (2016). Rheology of the active cell cortex in mitosis. Biophys. J..

[bib17] Garcia P.D., Garcia R. (2018). Determination of the viscoelastic properties of a single cell cultured on a rigid support by force microscopy. Nanoscale.

[bib18] Garcia P.D., Guerrero C.R., Garcia R. (2020). Nanorheology of living cells measured by afm-based force–distance curves. Nanoscale.

[bib19] Garcia R. (2020). Nanomechanical mapping of soft materials with the atomic force microscope: methods, theory and applications. Chem. Soc. Rev..

[bib20] Glaubitz M., Medvedev N., Pussak D., Hartmann L., Schmidt S., Helm C.A., Delcea M. (2014). A novel contact model for afm indentation experiments on soft spherical cell-like particles. Soft Matter.

[bib21] Guck J., Ananthakrishnan R., Moon T.J., Cunningham C.C., Käs J. (2000). Optical deformability of soft biological dielectrics. Phys. Rev. Lett..

[bib22] Hertz H. (1881). Über die berührung fester elastischer körper. J. Die Reine Angew. Math..

[bib23] Hochmuth R.M. (2000). Micropipette aspiration of living cells. J. Biomech..

[bib24] Hutter J.L., Bechhoefer J. (1993). Calibration of atomic-force microscope tips. Rev. Scientific Instr..

[bib25] Kalcioglu Z.I., Mahmoodian R., Hu Y., Suo Z., Van Vliet K.J. (2012). From macro-to microscale poroelastic characterization of polymeric hydrogels via indentation. Soft Matter.

[bib26] Kuznetsova T.G., Starodubtseva M.N., Yegorenkov N.I., Chizhik S.A., Zhdanov R.I. (2007). Atomic force microscopy probing of cell elasticity. Micron.

[bib27] Lautenschläger F., Paschke S., Schinkinger S., Bruel A., Beil M., Guck J. (2009). The regulatory role of cell mechanics for migration of differentiating myeloid cells. Proc. Natl. Acad. Sci..

[bib28] Lee E.H., Radok J.R.M. (1960). The contact problem for viscoelastic bodies. J. Appl. Mech..

[bib29] López-Guerra E.A., Solares S.D. (2014). Modeling viscoelasticity through spring–dashpot models in intermittent-contact atomic force microscopy. Beilstein J. Nanotechnol..

[bib30] Mahaffy R.E., Shih C.K., MacKintosh F.C., Käs J. (2000). Scanning probe-based frequency-dependent microrheology of polymer gels and biological cells. Phys. Rev. Lett..

[bib31] Mathur A.B., Collinsworth A.M., Reichert W.M., Kraus W.E., Truskey G.A. (2001). Endothelial, cardiac muscle and skeletal muscle exhibit different viscous and elastic properties as determined by atomic force microscopy. J. Biomech..

[bib32] Mchedlishvili N., Matthews H.K., Corrigan A., Baum B. (2018). Two-step interphase microtubule disassembly aids spindle morphogenesis. BMC Biol..

[bib33] Müller P. (2019). Pyjibe (0.13.2). https://github.com/AFM-analysis/PyJibe.

[bib34] Mokbel D., Abels H., Aland S. (2018). A phase-field model for fluid-structure-interaction. J. Comput. Phys..

[bib35] Mokbel M., Hosseini K., Aland S., Fischer-Friedrich E. (2020). The poisson ratio of the cellular actin cortex is frequency dependent. Biophys. J..

[bib36] Möllmert S., Kharlamova M.A., Hoche T., Taubenberger A.V., Abuhattum S., Kuscha V., Kurth T., Brand M., Guck J. (2020). Zebrafish spinal cord repair is accompanied by transient tissue stiffening. Biophys. J..

[bib37] Moreno-Flores S., Benitez R., dM Vivanco M., Toca-Herrera J.L. (2010). Stress relaxation and creep on living cells with the atomic force microscope: a means to calculate elastic moduli and viscosities of cell components. Nanotechnology.

[bib38] Moshayedi P., Costa L.D.F., Christ A., Lacour S.P., Fawcett J., Guck J., Franze K. (2010). Mechanosensitivity of astrocytes on optimized polyacrylamide gels analyzed by quantitative morphometry. J. Phys. Condens. Matter.

[bib39] Müller P., Abuhattum S., Möllmert S., Ulbricht E., Taubenberger A.V., Guck J. (2019). nanite: using machine learning to assess the quality of atomic force microscopy-enabled nano-indentation data. BMC Bioinf..

[bib40] Nawaz S., Sánchez P., Bodensiek K., Li S., Simons M., Schaap I.A. (2012). Cell visco-elasticity measured with afm and optical trapping at sub-micrometer deformations. PLoS One.

[bib41] Niu T., Cao G. (2014). Finite size effect does not depend on the loading history in soft matter indentation. J. Phys. D: Appl. Phys..

[bib42] Otto O., Rosendahl P., Mietke A., Golfier S., Herold C., Klaue D., Girardo S., Pagliara S., Ekpenyong A., Jacobi A. (2015). Real-time deformability cytometry: on-the-fly cell mechanical phenotyping. Nat. Methods.

[bib43] Rebelo L.M., de Sousa J.S., Mendes Filho J., Radmacher M. (2013). Comparison of the viscoelastic properties of cells from different kidney cancer phenotypes measured with atomic force microscopy. Nanotechnology.

[bib44] Rother J., Nöding H., Mey I., Janshoff A. (2014). Atomic force microscopy-based microrheology reveals significant differences in the viscoelastic response between malign and benign cell lines. Open Biol..

[bib45] Rusaczonek M., Zapotoczny B., Szymonski M., Konior J. (2019). Application of a layered model for determination of the elasticity of biological systems. Micron.

[bib46] Sanchez J.G., Espinosa F.M., Miguez R., Garcia R. (2021). The viscoelasticity of adherent cells follows a single power-law with distinct local variations within a single cell and across cell lines. Nanoscale.

[bib47] Sokolov I., Iyer S., Subba-Rao V., Gaikwad R.M., Woodworth C.D. (2007). Detection of surface brush on biological cells in vitro with atomic force microscopy. Appl. Phys. Lett..

[bib48] Steigemann P., Wurzenberger C., Schmitz M.H., Held M., Guizetti J., Maar S., Gerlich D.W. (2009). Aurora b-mediated abscission checkpoint protects against tetraploidization. Cell.

[bib49] Taubenberger A.V., Baum B., Matthews H.K. (2020). The mechanics of mitotic cell rounding. Front. Cell Dev. Biol..

[bib50] Ting T. (1966). The contact stresses between a rigid indenter and a viscoelastic half-space. J. Appl. Mech..

[bib51] Vey S., Voigt A. (2007). AMDiS: adaptive multidimensional simulations. Comput. Visual. Sci..

[bib52] Virtanen P., Gommers R., Oliphant T.E., Haberland M., Reddy T., Cournapeau D., Burovski E., Peterson P., Weckesser W., Bright J. (2020). SciPy 1.0: fundamental algorithms for scientific computing in python. Nat. Methods.

[bib53] Witkowski T., Ling S., Praetorius S., Voigt A. (2015). Software concepts and numerical algorithms for a scalable adaptive parallel finite element method. Adv. Comput. Math..

[bib54] Zhang J., Ru J., Chen H., Li D., Lu J. (2017). Viscoelastic creep and relaxation of dielectric elastomers characterized by a kelvin-voigt-maxwell model. Appl. Phys. Lett..

