# Supplementary material for: An explicit model to extract viscoelastic properties of cells from AFM force-indentation curves
Source: iScience. 2022 Mar 5;25(4):104016. doi: 10.1016/j.isci.2022.104016 (PMC8931349; doi:10.1016/j.isci.2022.104016)
Supplement: Document S1. Figures S1–S6 [file mmc1.pdf]

iScience, Volume *25*

Supplemental information

An explicit model to extract viscoelastic properties of cells from AFM force-indentation curves

Shada Abuhattum, Dominic Mokbel, Paul Müller, Despina Soteriou, Jochen Guck, and Sebastian Aland

Supplementary information

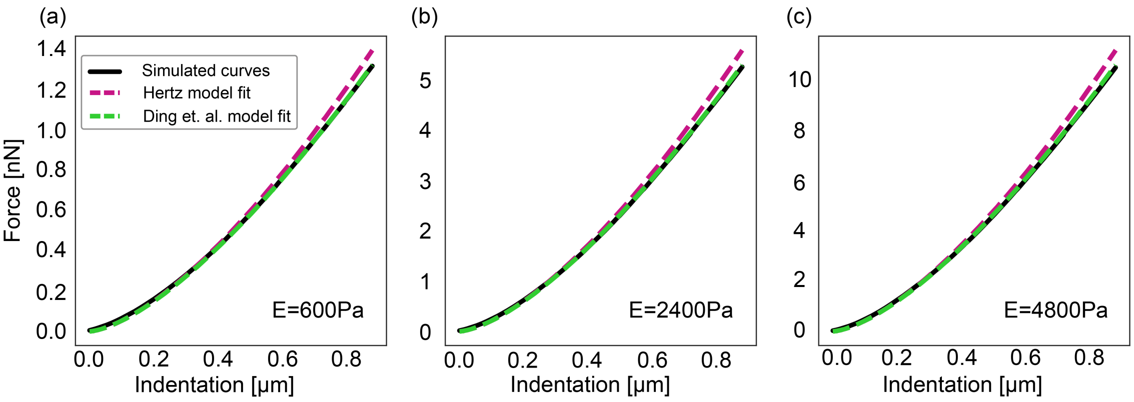

Figure S1: Validation of the numerical model, related to Fig. 1. Simulating a model with purely elastic material having different elasticity values : (a) 600 Pa, (b) 2400 Pa and (c) 4800 Pa. The black continuous line depicts the force indentation curve retrieved from the indentation simulation. The pink and green dashed lines correspond to the Hertz and Ding et al. models described in eq. (1) and (2) in the manuscript using the same value of the simulated elastic modulus.

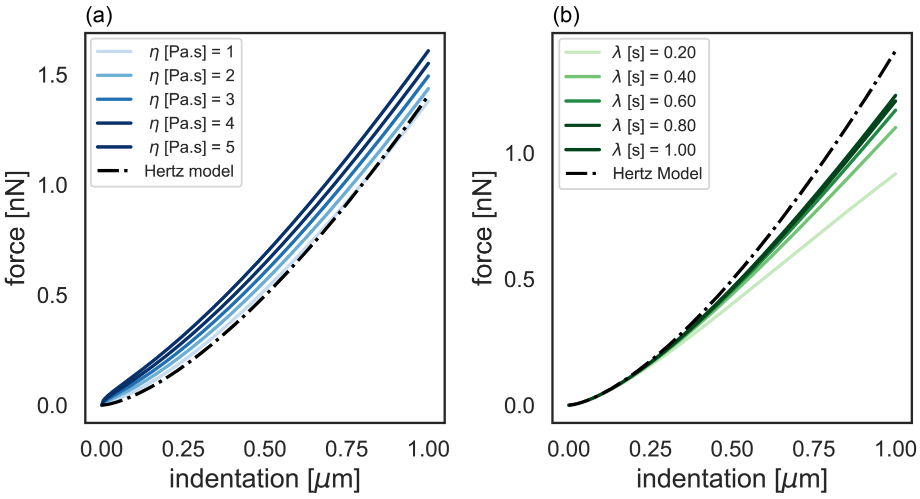

Figure S2: Force evolution during the indentation, related to Fig. 2. (a) Force evolution of $F = F_{elastic}(E_0, \delta) + \alpha_2 \delta^{\alpha_3} R^{\alpha_4} \eta \mathrm{v}$ in eq. (3) in the manuscript, where $E_0 = 500$ Pa and the viscosity η was varied between $1 - 5$ Pa.s. (b) Force evolution of $F = F_{elastic}(E_1, \delta) \cdot \exp(\frac{-\alpha_1 \delta}{\mathrm{v}\lambda})$ in eq. (3) in the manuscript, where $E_1 = 500$ Pa and the relaxation time λ was varied in the range of $0.2 - 1$ s. The black line depicts the force–indentation of the Hertz model with a Young's Modulus of 500 Pa. For all the subfigures, the radius of the indenter is $R = 2.5$ μm, the Poisson's ration $\nu = 0.5$ and the velocity is $\mathrm{v} = 5$ μm/s.

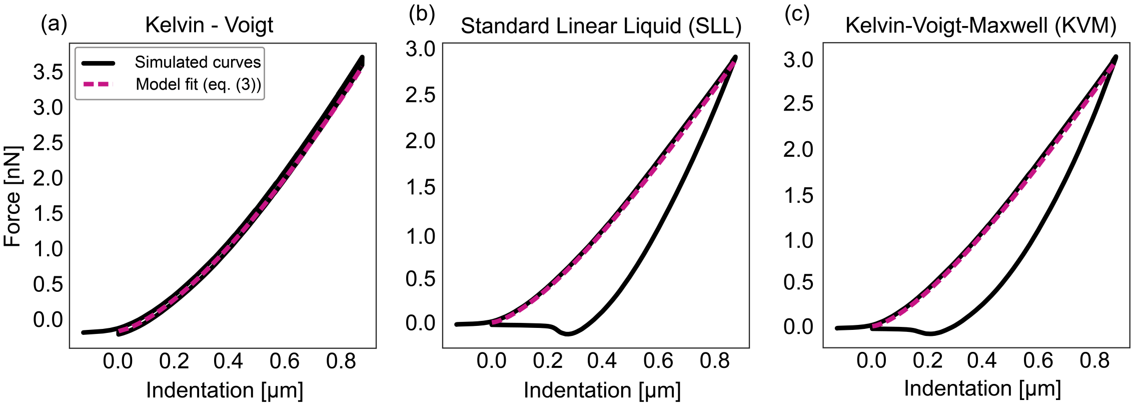

	Simulation	Fit
E_0 [Pa]	1650	1597
E_1 [Pa]	0	0
λ [s]	-	-
η [Pa.s]	0.5	0.57
E_u [Pa]	1650	1597
E_{app} [Pa]	1650	1597

	Simulation	Fit
E_0 [Pa]	0	100
E_1 [Pa]	1650	1410
λ [s]	0.325	0.38
η [Pa.s]	0.1	0.13
E_u [Pa]	1650	1510
E_{app} [Pa]	1354	1290

	Simulation	Fit
E_0 [Pa]	900	850
E_1 [Pa]	900	775
λ [s]	0.1	0.14
η [Pa.s]	0.1	0.14
E_u [Pa]	1800	1625
E_{app} [Pa]	1373	1340

Figure S3: Validation of the fitting model, related to Fig. 1 and Fig. 2. Fitting the model in eq. (3) to simulated force–indentation curves with different material properties; (a) Kelvin–Voigt, (b) standard linear liquid and (d) Kelvin–Voigt–Maxwell. The black line depicts the force–indentation curves retrieved from the simulation. The pink dashed line depicts the model fit. The tables below each curve show the simulated and fitted values of the mechanical parameters.

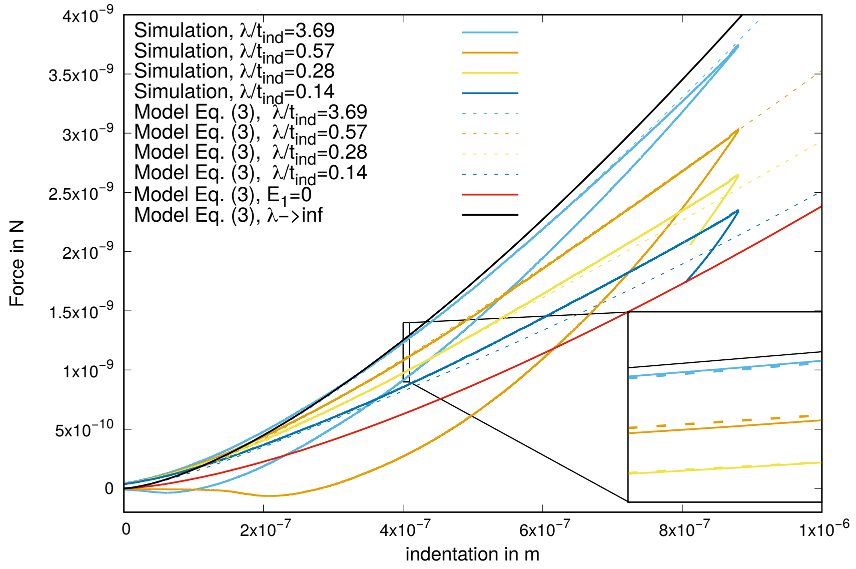

Figure S4: Validity of the fitting model in different parameter ranges, related to Fig. 1 and Fig. 2. Simulating different values for Maxwell relaxation λ. The ratio of the Maxwell relaxation and the indentation time λ/t_{ind} is shown in the figure. The parameters used for the simulations are, indentation time $t_{\text{ind}} = 0.176$ s, v $= 5$ µm/s $E_0 = E_1 = 900$ Pa, $R = 2.5$ µm.

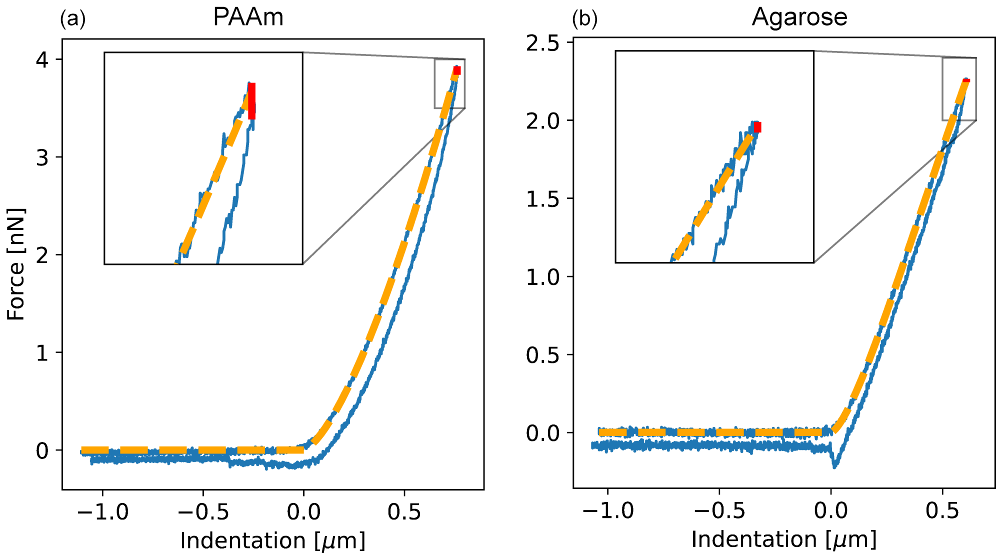

	Fit
E_0 [Pa]	2142
E_1 [Pa]	0
λ [s]	-
η [Pa.s]	1.1
E_u [Pa]	2142
E_{app} [Pa]	2142

	Fit
E_0 [Pa]	434
E_1 [Pa]	2181
λ [s]	0.09
η [Pa.s]	0.38
E_u [Pa]	2621
E_{app} [Pa]	1714

Figure S5: Representative force-indentation curves, related to Fig. 3 and Fig. 4. Force-indentation curves of (a) PAAm and (b) Agarose hydrogels. The blue line shows a representative force-indentation curve acquired by the AFM, the dashed orange line shows the fitting model in eq. (3) in the main manuscript where the fitted parameters are shown in the table below each figure, and the vertical red line highlights the jump at the end of the approach and the beginning of the retraction curve. For these measurements, the radius of indenter is $R = 2.5$ µm and the indentation velocity is v $= 5$ µm/s.

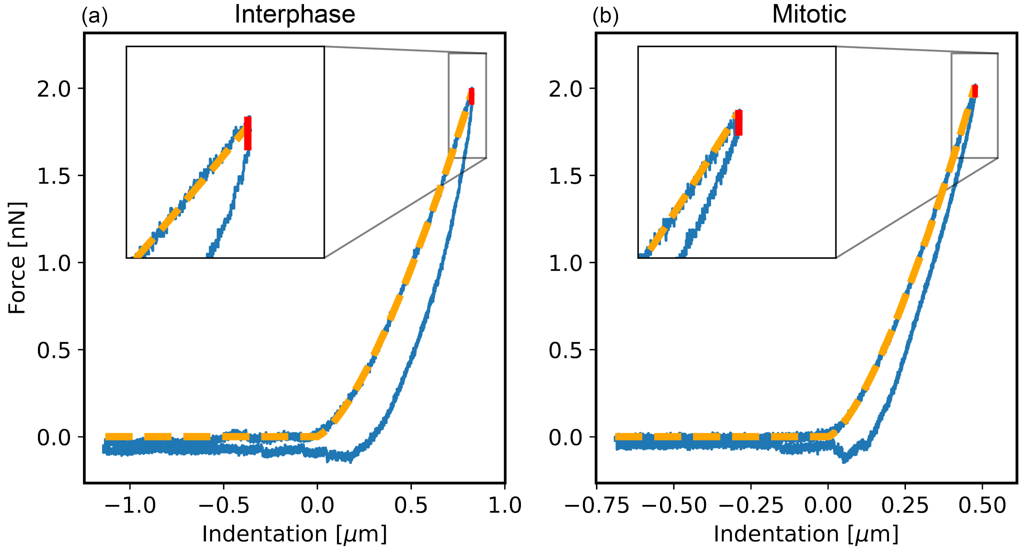

	Fit
E_0 [Pa]	966
E_1 [Pa]	0
λ [s]	-
η [Pa.s]	2.3
E_u [Pa]	966
E_{app} [Pa]	966

	Fit
E_0 [Pa]	2404
E_1 [Pa]	299
λ [s]	0.18
η [Pa.s]	1.6
E_u [Pa]	2703
E_{app} [Pa]	2535

Figure S6: Representative force-indentation curves, related to Fig. 5. Force-indentation curves of (a) interphase and (b) mitotic HeLa cells. The blue line shows a representative force-indentation curve acquired by the AFM, the dashed orange line shows the fitting model in eq. (3) in the main manuscript where the fitted parameters are shown in the table below each figure, and the vertical red line highlights the jump at the end of the approach and the beginning of the retraction curve. For these measurements, the radius of indenter is $R = 2.5$ μm and the indentation velocity is $v = 2$ μm/s.
